# A systematic review on the bidirectional relationship between trauma-related psychopathology and reproductive aging

**DOI:** 10.1016/j.xjmad.2024.100082

**Published:** 2024-08-29

**Authors:** Amanda R. Arnold, Trinidi Prochaska, Maximilian Fickenwirth, Abigail Powers, Alicia K. Smith, E. Britton Chahine, Jennifer S. Stevens, Vasiliki Michopoulos

**Affiliations:** a Department of Psychiatry and Behavioral Sciences, Emory University School of Medicine, Atlanta, GA, United States; b Department of Gynecology and Obstetrics, Emory University School of Medicine, Atlanta, GA, United States

**Keywords:** Trauma, PTSD, Depression, Menopause

## Abstract

**Objective:**

Natural variation in ovarian steroid hormones across the female lifespan contributes to an increased risk for depressive and posttraumatic stress disorder (PTSD) symptoms in women. However, minimal work has focused on understanding the impacts of reproductive aging on the brain and behavioral health of trauma-exposed women. This systematic review examines the bidirectional relationship between trauma-related psychopathology and reproductive aging.

**Method:**

Following PRISMA guidelines, a systematic review of PubMed, PsychInfo, and Medline databases was undertaken to identify controlled studies on how trauma history impacts psychopathology and menopause symptoms during reproductive aging.

**Results:**

Twenty-one studies met the eligibility criteria, with only four utilizing the gold standard STRAW+ 10 criteria for defining reproductive aging stages. The peri and postmenopausal periods appear to be particularly vulnerable phases for individuals with trauma exposure. Menopause symptoms and trauma-related psychopathology symptom severity increase during reproductive aging with increases in the degree of trauma exposure. However, mechanistic insights that may explain this interaction are currently neglected in this area of research.

**Conclusion:**

There is a significant lack of understanding regarding how reproductive aging and its related neuroendocrine changes impact the brain to influence PTSD and depression symptoms related to trauma exposure. This lack of basic understanding impedes the ability to identify, assess, and treat PTSD and depressive symptoms in trauma-exposed women most effectively, and mitigate the long-term consequences of these behavioral health symptoms on morbidity and mortality in aging women.

## 1. Introduction

Seventy to ninety percent of people will experience at least one traumatic event in their lifetime [1]. Trauma exposure can result in chronic mental health conditions that increase the risk for the development of chronic morbidity and mortality [2–6]. Depression and posttraumatic stress disorder (PTSD) are among the most common reactions to trauma [7,8]. Importantly, depression and PTSD are more than twice as prevalent in women than men [9–13]. Women have increased exposure to specific types of traumatic events, including sexual assault, and have greater prevalence of comorbid psychiatric disorders compared to men [9,14,15]. However, these differences do not fully account for the overall sex difference in the prevalence of depression and PTSD in individuals who have experienced trauma [16].

Accumulating translational evidence from reproductive aged women indicates that natural variation in ovarian steroid hormones (e.g., estradiol and progesterone) over the course of the menstrual cycle and in the postpartum period contribute to an increased risk for depressive and PTSD symptoms in women [17–23] Estradiol withdrawal that occurs in the luteal phase of the menstrual cycle and in the postpartum period can impact the brain and exacerbate PTSD symptoms [24,25]. Decreasing levels of allopregnanolone, a progesterone-derived neurosteroid that declines in the late luteal phase of the menstrual cycle and in the postpartum period, have also been implicated in the etiology of depression and PTSD [26–28]. Although changes in estradiol and progesterone are linked to increased mood symptoms in premenopausal women [29–31], research to date has primarily focused on reproductive-aged women, with minimal attention to the menopause transition that is also characterized by robust changes in estradiol and progesterone levels [32].

Reproductive aging refers to the gradual decline in ovarian function and fertility throughout a woman’s life, culminating in menopause. In the current review, we use the term ‘reproductive aging’ to encompass all stages related to the menopause transition (perimenopause), menopause itself, and the postmenopausal period. The Stages of Reproductive Aging Workshop + 10 (STRAW+10) criteria are recognized as the gold standard for characterizing reproductive aging and provide a comprehensive framework for assessing stages through the menopause transition and into postmenopause [33]. According to STRAW+ 10, the menopause transition begins with the onset of menstrual cycle irregularity and concludes with the final menstrual period (FMP). This transition includes early and late stages of perimenopause, characterized by increased variability in the intervals between menstrual cycles (>7 day different from normal) and periods of amenorrhea. The occurrence of menopause is defined as 12 months of amenorrhea and marks the end of the menopause transition. The postmenopausal period that follows is defined as the time after this 12-month mark [33]. Throughout the menopause transition, women experience erratic fluctuations in estradiol and progesterone as ovarian function declines. These changes in estradiol and progesterone occur alongside increases in follicle-stimulating hormone (FSH) and decreases in Anti-Mullerian Hormone (AMH) [32,33] and have been linked to various symptoms of reproductive aging, including vasomotor symptoms, sleep disturbances, sexual dysfunction, and cognitive changes [34,35], however the specific mechanisms and factors that confer risk for trauma-related mood disruptions during reproductive aging remain unclear.

Despite evidence showing that changes in mood symptoms during perimenopause are linked to increased risk for all-cause mortality in women [36,37], minimal research has directly investigated the bidirectional associations between trauma-related psychopathology and reproductive aging. Here, we conducted a systematic review to address this gap. We examined the intersection of trauma-related psychopathology and reproductive aging in trauma-exposed individuals. Our objectives were to systematically identify existing evidence linking 1) reproductive aging to risk for trauma-related psychopathology, focusing on PTSD and depression; 2) trauma and PTSD to menopause onset and symptoms, including effects on cognitive and brain function; and 3) to identify gaps in our understanding of how reproductive aging impacts trauma-related psychopathology and vice versa, and the underlying biological mechanisms by which this occurs. Increasing our basic knowledge about how biological changes during reproductive aging impact the brain to influence mental health is critical for the identification, assessment, and treatment of PTSD and depressive symptoms in women who have experienced trauma. By synthesizing insights from the available literature, we aim to guide future research and clinical practice to ultimately mitigate the long-term consequences of trauma exposure on women’s health during reproductive aging.

## 2. Methods

The current systematic review of the literature was performed according to PRISMA systematic review guidelines [38]. Utilizing PubMed, PsychInfo, and Medline databases, we searched in the title/abstract field employing the terms “Menopause” OR “Menopausal.” Each search incorporated one of the following additional terms: “Posttraumatic Stress Disorder,” “Trauma Exposure,” “Interpersonal Violence,” “Domestic Violence,” “Sexual Assault,” “Sexual Trauma,” “Adverse Childhood Experiences,” “Childhood Trauma,” and “Childhood Abuse.” Articles were chosen based on predefined inclusion and exclusion criteria. Eligibility criteria deemed a study suitable if it presented original data about women undergoing the menopausal transition, with a clearly defined measure of trauma exposure. Only studies assessing the impact of reproductive aging on trauma-related mental health outcomes and menopause symptom severity in individuals with a trauma history were included. Reviews, opinion pieces, case reports, and letters to the editor were excluded from consideration. One reviewer (TP) searched using the terms cited above in the eligibility criteria to identify relevant studies. This same reviewer (TP) screened titles and abstracts to remove duplicates. Then titles and abstracts were assessed independently by two reviewers (TP and AA) and articles were excluded if both reviewers decided that the articles did not meet the criteria. In case of disagreement, the two reviewers had to reach a consensus. Then, the same two reviewers assessed the full-text versions of the remaining articles independently against inclusion and exclusion criteria. In case of disagreement, the two reviewers had to reach a consensus.

## 3. Results

**Fig. 1.**
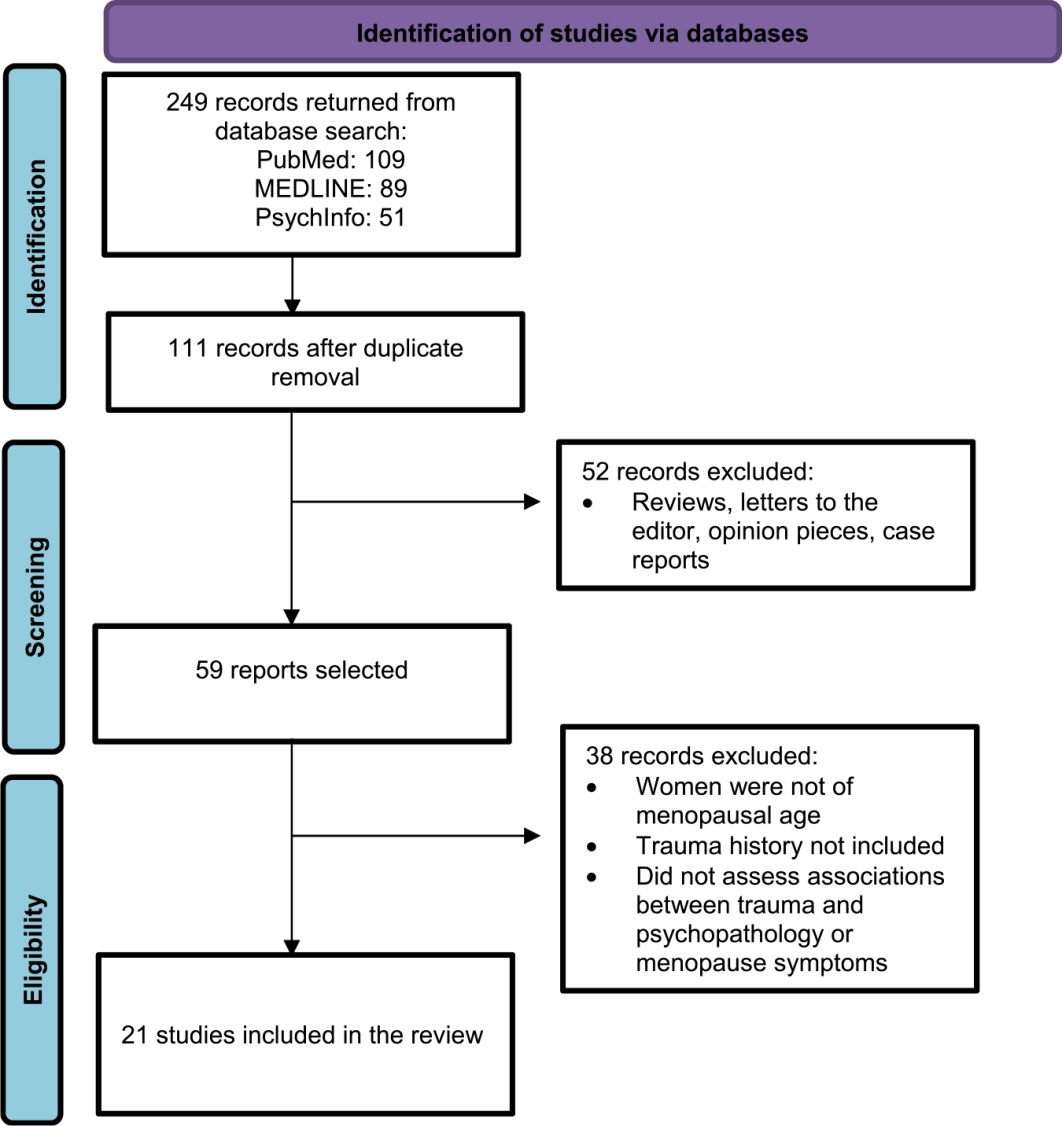
Summary of literature search Adapted from PRISMA [38].

The initial search on PubMed, PsycInfo, and Medline was conducted from January 2024 to April 2024, and provided 249 potentially eligible studies. Once duplicates had been removed, a total of 111 titles and abstracts were screened. We excluded 52 articles because they were not primary data manuscripts. After full text reviews of the 59 remaining reports, 38 were excluded as they did not assess women of menopausal age, trauma history, and the impact of trauma on psychopathology or menopause symptoms (Fig. 1). Of the 21 studies that met inclusion criteria, 8 focused on depression in trauma-exposed women of menopausal age, 3 on PTSD in trauma-exposed women of menopausal age, and 15 on the effects of trauma and PTSD on menopause onset and symptoms, including cognitive function, in trauma-exposed women of menopausal age (Tables 1 and 2). It’s important to note that some studies contributed findings to more than one of these categories, reflecting the interconnected nature of these outcomes in trauma exposed women during reproductive aging. In addition, only four out of these 21 studies explicitly used the STRAW+ 10 criteria to define menopausal stages of reproductive aging (Tables 1 and 2). The remaining studies used various methods, including self-reported menstrual patterns, age-based estimates, or did not clearly specify their assessment methods.

### 3.1. Increased depression in trauma-exposed women during reproductive aging

Results from our systematic review suggest that women of menopausal age, who experienced trauma at various points in their lives, are more susceptible to depression than those who do not experience trauma (Table 1).

#### Childhood trauma.

There has been a particular focus on the effects of exposure to childhood trauma, including maltreatment and adverse childhood experiences (ACES) on depression and depressive symptoms during reproductive aging. Shea et al. conducted a cross-sectional study that revealed that greater exposure to childhood maltreatment were associated with more severe depressive symptoms in postmenopausal women [39]. Metcalf et al. found that both early child adversity and current life stressful events predicted an increased likelihood of clinically significant depression in women of menopausal age within their longitudinal study [40]. Epperson et al., whom also conducted a longitudinal study, found that a history of childhood adversity (≥2 ACEs) and adolescent/adult adversity (≥2 ACEs after menarche) increased risk of a first major depressive episode during the menopause transition [41].

#### Adulthood trauma.

In addition to risk due to early adverse childhood experiences, three papers from our systematic review presented findings that support the notion that trauma exposure in adulthood also increases depressive symptoms throughout reproductive aging. Moraes et al. found within their cross-sectional study that peri and postmenopausal women with histories of domestic or sexual violence had significantly more comorbid health conditions, both physical and psychological, compared to non-abused controls, with depression being the most common comorbidity (69.4%) [42]. In addition, two cross-sectional studies of female veterans of menopausal age with a history of military sexual trauma (MST) showed an increased likelihood of meeting diagnostic criteria for depression compared to female veterans without MST history [43,44].

An open question is whether the association of trauma and depressive symptoms is specific to peri or postmenopause, or whether a history of trauma increases symptom burden in a uniform way across the lifespan. Michopoulos et al. conducted a cross-sectional study that found that trauma-exposed women of perimenopausal age reported higher levels of depressive symptoms than pre and postmenopausal aged women, regardless of the timing of trauma exposure (e.g., trauma exposure as a child versus as an adult) [45]. This may vary depending on whether women were already experiencing significant depressive symptoms prior to entering the menopausal transition. For example, [46] found in their longitudinal study that odds of a major depressive episode (MDE) were greater during perimenopause and postmenopause only in women without prior MDD. However, in women with a history MDD, childhood maltreatment interacted with menopause status to increase MDE risk only in postmenopausal women [46].

### 3.2. Increased PTSD in trauma-exposed women during reproductive aging

Although trauma exposure and PTSD are often co-morbid with depression [47], the effects of reproductive aging on trauma-related symptoms of PTSD have received minimal research focus to date. We identified only three studies that directly examined this topic (Table 1). Michopoulos et al. found that perimenopausal aged women reported higher levels of PTSD symptoms compared to those in premenopausal and postmenopausal aged women [45]. Further, two studies of female veterans of menopausal age with an MST history showed an increased likelihood of meeting the criteria for a PTSD diagnosis compared to female veterans without a history of MST [43,44]. This aligns with the data mentioned in Section 3.1 indicating more severe depression during reproductive aging, suggesting an increased susceptibility to trauma-related psychopathology during reproductive aging.

### 3.3. Trauma and PTSD effects on menopause onset and symptoms

Most of the studies identified by our systematic review examined the effects of trauma and/or PTSD on menopause onset and symptom severity, including impacts on cognitive function (Table 2). In our systematic review, we found 15 studies that investigated the impact of trauma exposure and/or PTSD on menopause timing and onset, menopause symptom severity, and cognition and brain function.

#### Timing of menopause.

Three studies investigated the timing of menopause in trauma-exposed women, with mixed results. Mendoza-Huertas et al. (2024) found in their cross-sectional study that violence-exposed women reached menopause approximately 20 months earlier and 20.7% of these women developed premature ovarian insufficiency [48]. Alternatively, Nishimi et al. and Kling et al., both concluded that time to natural cessation of menses was not significantly different between trauma-exposed and no-trauma groups [49,50]. However, Nishimi et al. found in their longitudinal study, which included a sample size of 46,639 people, that trauma-exposed individuals were more likely to have earlier cessation of menses due to gynecological surgeries, a factor that was not considered in the Mendoza-Huertas study.

#### Menopause symptom severity.

Across the eight studies examining trauma exposure among women of menopausal age, there is consistent evidence linking trauma history, including ACEs, intimate partner violence, and sexual trauma to greater overall menopausal symptom burden (Table 2). Most investigated the impact on trauma history on menopause symptom severity broadly [39,42,48,51,52], one examined sexual function specifically [53], two investigated vasomotor symptoms specifically [54,55], and one investigated sleep symptoms specifically [56]. Associations were found between trauma exposure and higher total scores on menopausal symptom measures like the Kupperman Menopausal Index [42], Greene Climacteric Scale [39], Cerventes Scale [48] and Menopause Rating Scale [52]. Notably, women who experienced abuse in both childhood/adolescence and adulthood had the highest scores on the menopause symptom questionnaire [42].

#### Cognitive and brain function.

Four studies examined the impact of ACEs on cognitive and brain function in menopausal aged women (Table 2). Metcalf et al. (2022) and Shanmugan et al. (2017a) found that women with high ACE scores showed poorer performance on objective cognitive tests during the menopausal transition [57–59]. Metcalf et al. (2022) conducted a longitudinal study and found that childhood adversity interacted with inflammatory markers to predict worse verbal memory performance during perimenopause [57]. Similarly, Shanmugan et al. (2017a) found in their cross-sectional study that high childhood adversity was associated with worse performance on tests of sustained attention and working memory in menopausal women [59]. These deficits in attention and working memory linked to childhood adversity were also associated with decreased activation in brain regions supporting executive function [58], lower functional connectivity within and between executive control networks [59], and reduced network flexibility [60] in women of menopausal age.

## 4. Discussion

The current systematic review identified 21 studies that have focused on the impacts of reproductive aging on risk for trauma-related psychopathology, focusing on PTSD and depression and the impacts of trauma exposure and PTSD on menopause onset and symptoms, including effects on cognitive and brain function. We found considerable variation in how reproductive aging stages were defined and assessed. Only four studies utilized the standardized STRAW+ 10, while the remaining studies used a range of other methods. These methods included self-reported menstrual patterns, age-based estimates, and in some cases, the assessment methods were not clearly specified. Furthermore, only three studies compared symptoms of menopause or trauma-related psychopathology across different stages of reproductive aging. Despite the failure of the majority of existing studies to utilize a reliable definition of menopausal status in the reviewed articles, the existing studies provide evidence for a bidirectional relationship between trauma-related psychopathology and reproductive aging. Our review not only synthesizes the current knowledge but also highlights the critical need for the field to assess and use the STRAW+ 10 criteria in future research to enhance our understanding of how different stages of reproductive aging may differentially impact trauma-related psychopathology.

The minimal data to date from eight studies indicate reproductive aging can impact depression and PTSD symptoms in trauma-exposed women [39–46]. In addition, women with a history childhood maltreatment and sexual trauma appear to be at heightened risk for the emergence or exacerbation of PTSD and depression during reproductive aging [39–41,43,44,46]. Although this vulnerability is reflected in the increased prevalence and severity of depression and PTSD symptoms compared to non-trauma-exposed women, the effects of reproductive aging on these symptoms of trauma-related psychopathology were sometimes apparent in perimenopausal [45] or postmenopausal women [39,42,44,48,50,51,56], or both [46]. These findings suggest that variation in estradiol and progesterone over the course of reproductive aging may contribute to an increased risk for depressive and PTSD symptoms in trauma-exposed women, similar to what is seen during the menstrual cycle and in the postpartum period [17–23,26–31].

Published findings from three studies identified by our systematic review described the impact of trauma exposure and/or PTSD on menopause timing. Although one report indicated that trauma exposure was associated with earlier age at menopause [48], two other studies concluded that time to natural cessation of menses was not significantly different due to trauma exposure [49,50]. However, one of those studies reported that trauma-exposed individuals were more likely to have earlier cessation of menses due to gynecological surgeries [49]. These minimal findings linking trauma to reproductive senescence aligns with existing research that connects PTSD to an increased risk of gynecological complications, such as fibroids, endometriosis, and polycystic ovarian syndrome, which often necessitate gynecological surgeries [51,61]. Further, each of these three studies examined the effects of trauma at different timepoints in life on menopause timing with Mendoza-Huertas et al. (2024) examining partner violence in adulthood, [49] examining lifetime trauma and PTSD symptomology, and Kling et al. examining ACEs. Further studies are needed to delineate if timing of trauma exposure has differential effects on menopause onset or risk for the development of gynecological conditions.

Eight studies identified by our systematic review linked various types of trauma exposure, including ACEs, intimate partner violence, and sexual trauma, to greater overall menopause symptom burden [39,42,51,52], including sexual function [53], vasomotor symptoms [54,55] and sleep symptoms [56]. An additional four studies examined the impact of ACEs on cognitive and brain function in menopausal aged women [57–60]. These studies showed that high childhood adversity was associated with worse performance on tests of sustained attention and working memory, as well as with decreased activation in brain regions supporting executive function [58], lower functional connectivity within and between executive control networks [59], and reduced network flexibility [60].

The studies identified by our systematic review had several strengths, including the use of validated measures of trauma exposure, depression and PTSD symptoms, the inclusion of diverse trauma types and assessment of their unique contribution to trauma-related psychopathology, and the examination of multiple mental and physical health outcomes that are impacted by the reproductive aging. It’s important to note that while our review focused on trauma-related psychopathology, there is strong evidence from large longitudinal studies, such as the Study of Women’s Health Across the Nation (SWAN), the Personality and Total Health (PATH) Through Life Project, and the Harvard Study of Moods and Cycles demonstrating increased risk for depression during reproductive aging outside the context of trauma exposure [62–64]. This vulnerability during reproductive aging is also highlighted by other studies showing that hormonal changes during the menopausal transition are associated with increased risk of depressive symptoms even in women with no history of depression [65].

The susceptibility during reproductive aging extends beyond psychological health, as vasomotor symptoms during reproductive aging are associated with increased cardiovascular risk [66] and higher fracture incidence [67]. Overall existing studies suggest that the peri and postmenopausal periods represent a unique phase of vulnerability for various negative health outcomes, even in the absence of trauma exposure. However, comparable large-scale longitudinal data for PTSD as an outcome during reproductive aging is lacking, despite the fact that biological factors that influence PTSD symptoms, such as inflammation [68] and hormonal fluctuations [22] also contribute to health vulnerabilities of reproductive aging [69]. Indeed, inflammation and activity of the immune system are modulated by ovarian hormones [70–74] and serum cytokine concentrations are associated PTSD [68] and psychological symptoms in midlife women [75]. In addition, hormonal fluctuations, including changes in neurosteroid levels, and HPA axis dysregulation are linked to PTSD [22]and to perimenopausal depression [30].

Several limitations of the existing literature should be noted. First, our review revealed significant inconsistency in how menopause is defined and assessed across studies. Out of 21 studies included, only four (Epperson et al., 2017; Metcalf et al., 2022; Metcalf et al., 2023; Carson and Thurston 2019) explicitly used the standardized STRAW+ 10 criteria to define menopausal stages [40,41,55,57]. The remaining studies employed various methods, including self-reported menstrual patterns, age-based estimates, or did not clearly specify their assessment methods. This variability presents a significant challenge in interpreting and comparing results across studies. Moreover, only three studies compared symptoms of menopause or trauma-related psychopathology across menopause stages [45,46,57]. Most of the identified studies treated menopause status as a confounding factor, eliminating the opportunity to examine the role of the hormonal transition in affecting symptom severity directly. Comparing outcomes across menopause stages, ideally within longitudinal cohorts, is necessary to assess whether the transition represents a particularly vulnerable period for trauma-exposed women.

The examination how changes in specific hormone levels (e.g., estradiol, progesterone, FSH, AMH) across reproductive aging is also necessary to better understand the mechanisms contributing to increased risk for depression and PTSD during this reproductive stage. While most studies identified by our review verified menopause status through cycle tracking, and few included hormone assessments of follicle stimulating hormone and estradiol to verify menopause stage, none used hormone levels as predictors of trauma-related psychopathology. The longitudinal assessment of these hormones is currently limited by our ability to quantify them on the dynamic time scale on which they fluctuate during the menopause transition. The development and implementation of nanobiosensors and other technologies that can constitutively measure estradiol and progesterone levels is critical for determining how the erratic and unpredictable fluctuations in these hormones during the menopause transition contribute to symptoms of trauma-related psychopathology and menopause symptom severity [76]. To address these limitations, future research should use the standardized STRAW+ 10 criteria for assessing menopausal status, clearly report assessment methods, conduct more longitudinal studies tracking women through different menopausal stages, and integrate biological markers alongside clinical assessments. In addition, studies should focus specifically on the perimenopause phase and encourage interdisciplinary collaboration between mental health researchers and reproductive endocrinologists to ensure accurate assessment and interpretation of menopausal status in the context of trauma-related psychopathology.

An additional limitation of the identified studies is the predominance of studies focused on childhood abuse and military sexual trauma as specific contributors to affective disturbances during reproductive aging. Future work should include a broader range of trauma types and systematically examine the role of developmental timing of exposure on perimenopausal mental health. Future research should also prioritize multi-modal and objective physiological assessments of vasomotor symptoms, sleep, cardiovascular function, and brain activity rather than relying solely on self-report measures of depression, PTSD, and menopause symptoms. Integrating objective physiological indices will provide a more comprehensive understanding of the mechanisms contribute to trauma-related psychopathology during reproductive aging.

Finally, most of the identified studies primarily feature White participants, highlighting a potential gap in understanding perimenopausal risk for increased trauma-related psychopathology or menopause symptoms among diverse racial and ethnic groups. The underrepresentation of diverse women in studies may exacerbate already existing health inequities in trauma-related adverse health outcomes, as minoritized Black women face elevated risks of trauma exposure and depression and PTSD over the lifespan [77–81]. In addition, research suggests that Black women may have higher estradiol levels during the menstrual cycle and perimenopausal transition than non-Black women [82,83], as well as a greater likelihood of experiencing severe vasomotor and depressive symptoms during perimenopause [84–86]. Despite the established evidence for greater risk for trauma-related psychopathology and menopause symptoms, these minoritized women remain underrepresented in research on women’s reproductive and mental health. To address this inequity, future studies should embrace more diverse sample populations to not only bridge critical knowledge gaps but also inform the development of tailored treatment and prevention strategies for mood disturbances during reproductive aging.

While the results of our systematic review highlight the existing limited data supporting the premise that depression and PTSD symptoms are impacted by reproductive aging and that trauma exposure and the presence of trauma-related psychopathology can impact menopause symptoms, the exact mechanisms underlying these associations remain to be fully characterized. The bidirectional relationship suggests common underlying mechanisms for both mood and menopause-related symptoms, including deficits in cognitive function. The fluctuations and relative decline in estradiol and allopregnanolone levels during the menopause transition may disrupt emotional regulation and cognitive function in ways that exacerbate trauma-related symptoms by influencing the activity of common neurocircuitry. Indeed, estradiol and progesterone modulate the activity of the amygdala, hippocampus, and prefrontal cortex [87–89], all regions important for emotional regulation and cognitive function [19,90–96]. These neuroendocrine hormones exert their effects by impacting neurotransmitter systems, including serotonin, dopamine, and GABA, which are implicated in both depression and PTSD when dysfunctional [97–99]. Alterations in the structure and functional connectivity of the amygdala, hippocampus, and prefrontal cortex are central to the pathophysiology of depression [100,101], PTSD [100,102,103], and deficits in executive function [104,105].

In addition to directly impacting the neurocircuitry contributing to affective and cognitive dysfunction, the estradiol and progesterone fluctuations during the menopause transition can also impact other physiological systems that can contribute to depression and PTSD symptoms in trauma-exposed women. Estradiol and progesterone modulate the activity of the hypothalamic pituitary adrenal (HPA) axis and influence glucocorticoid signaling [106–108], both of which contribute to the etiology of PTSD and depression [109,110]. Estradiol and progesterone can also impact inflammatory processes throughout the body, which can also contribute to depression and PTSD symptoms [68,111]. Moreover, these hormonal changes can affect autonomic nervous system (ANS) function, potentially altering heart rate variability and ANS responsivity, which are often dysregulated in trauma-exposed individuals with PTSD and depression [112,113]. Furthermore, because estradiol and progesterone can influence the activity of these systems independently, they also may modulate how these physiological systems interact with each other to contribute to trauma-related psychopathology. Together, these data indicate that neuroendocrine changes over reproductive aging can modulate the activity of multiple biological systems that can contribute to heightened risk for depression and PTSD in trauma-exposed women.

The collective findings from the studies identified have important implications for clinical practice and future research. Screening for trauma history and monitoring for the emergence of mental health symptoms during the menopause transition may help identify women at risk and facilitate timely intervention. Trauma-informed approaches to menopause care, which acknowledge the potential impact of prior trauma on current symptoms and treatment response, may improve outcomes for trauma-exposed women. It’s important to note that several effective treatments for traumatic stress exist and are likely to benefit women experiencing increased symptoms during reproductive aging. These include evidence-based psychotherapies such as cognitive behavioral therapy, cognitive processing therapy, prolonged exposure, and eye movement desensitization and reprocessing, as well as pharmacological interventions like selective serotonin reuptake inhibitors (SSRIs) [114]. In addition, evidence suggests estrogen may help alleviate PTSD symptoms [115]. This finding raises the possibility that hormone replacement therapy, commonly used for perimenopausal symptoms, might also benefit those with PTSD. Further, evidence suggests hormone therapy may interact with SSRIs, as studies show that hormone therapy increases the effectivity of SSRIs in the treatment of depression [116–118]. Adapting these existing evidence-based treatments to address the unique challenges of reproductive aging may enhance their efficacy for this population. Future studies should investigate the mechanisms by which biological changes during reproductive aging, including the role of specific hormone profiles, inflammatory markers, and brain changes, contribute to risk for new onset and exacerbation of depression and PTSD symptoms in trauma-exposed women. These studies are necessary to inform the development of novel interventions that address the unique needs of trauma-exposed women during this transitional period late in life.

It is noteworthy that many symptoms of menopause overlap with those of PTSD and depression, which presents both challenges and opportunities for future research. Key overlapping symptoms include sleep disturbances, mood changes, cognitive difficulties, fatigue, anxiety and autonomic dysfunction. This overlap necessitates careful consideration in future studies to disentangle the causal relationships between menopausal status, trauma exposure, and depression and PTSD symptom presentation. To address this, future research in this area could apply several innovative approaches. Longitudinal studies tracking symptoms from premenopause through postmenopause in both trauma-exposed and non-exposed women could help establish temporal relationships between hormonal changes, trauma history, and symptom onset. Ecological momentary assessment (EMA) could capture real-time symptom fluctuations in mood and their relationship to hormonal changes and menopause-related symptoms. Network analysis techniques could map symptom interactions over time, potentially revealing distinct patterns for menopause-related versus trauma-related depression and PTSD symptoms. Combining hormone measurements with other biological markers, including psychophysiology and neuroimaging, in comprehensive mechanistic studies could provide more nuanced models of symptom etiology. Experimental manipulations using hormone replacement therapy or trauma-focused interventions could observe differential effects on various symptoms. Qualitative research through in-depth interviews could provide insights into how women themselves differentiate between menopause-related and trauma-related symptoms. Advanced statistical techniques like machine learning could identify complex patterns in symptom presentation that may distinguish between reproductive aging and trauma-related origins. Finally, neuroimaging studies comparing brain activity patterns during symptom provocation in menopausal women with and without trauma histories could provide valuable insights. By employing these methods, researchers could begin to disentangle the complex relationships between menopausal symptoms and trauma-related psychopathology, leading to more targeted and effective interventions for this population. This approach would not only advance our understanding of the intersection between trauma and reproductive aging but also potentially improve clinical outcomes for affected women.

## 5. Summary and conclusions

In conclusion, trauma-exposed women appear uniquely susceptible to reemergence and exacerbation of PTSD, depression, cognitive changes, and physical symptoms during reproductive aging. While exact mechanisms require further characterization, neuroendocrine alterations likely sensitize women to adverse mental and physical health effects during this time. Screening for trauma history and mood/cognitive changes during perimenopause could enable earlier intervention to mitigate long-term consequences of affective disturbances during the menopause transition. While there remains a significant lack of research into this critical window of reproductive aging for trauma-impacted women’s health, the importance of understanding the interplay between PTSD, depression, and reproductive aging is underscored by recent federal initiatives. In 2023, the White House launched the Initiative on Women’s Health Research, which prioritizes research on women’s midlife health and menopause-related conditions [119]. Concurrently, the National Institutes of Health launched an agency-wide effort to close gaps in women’s health research across the lifespan, with a specific focus on the impact of reproductive aging on various health outcomes [119]. These initiatives highlight the growing recognition of the complex, multisystem effects of reproductive aging and the need for interdisciplinary research to identify mechanism that confer risk for adverse mental health outcomes. Furthering our basic understanding of how biological changes during this period impact the brain and behavior is essential for effectively identifying, assessing, and treating PTSD and depression in trauma-exposed women.

## Figures and Tables

**Table 1:** Summary of current literature evaluating the impact of trauma exposure and menopause status on PTSD, anxiety, and depression in menopausal women.

Reference	Sample/Age	Racial-Ethnic Demographics	Trauma Type/Independent Variable	Outcome Measure	Results	Assessment of Menopause Stage

[46]	Women (N = 333) interviewed annually over 15 years 42 to 52 years old	67 % White 33 % Black/African American	Current stress, CTQ status, menopause status, lifetime MDD diagnosis	Prevalence of MDEs	CM, but not current stress, interacted with menopausal status to increase risk for MD during postmenopause in women with MDD. Women without prior MDD were at increased risk for MD during peri and postmenopause.	Used data from the Study of Women’s Health Across the Nation (SWAN), which defined menopausal transition stages based on menstrual bleeding patterns
[42]	Women with one year of amenorrhea (N = 120) 40 to 65 years old	62.1 % White 37.1 % Black/African American 0.8 % Asian	Violence exposure status	Menopause symptom severity and rate of comorbid disorders such as osteoporosis, depression/psychological disorders, hypertension, rheumatic disease, allergies, fibromyalgia, varicose veins in the legs, labyrinthitis, diabetes, herniated disk, cancer	Women who experienced violence had a higher number of self-reported comorbidities during menopause (average of 4 –5) compared to 2.2 comorbidities in the control group. Total trauma exposure was associated with greater menopausal symptom severity. Sexual violence specifically was associated with poorer sexual health outcomes, but not with other menopausal symptoms.	Focused on postmenopausal women, defined as those who had their last menstrual period at least 12 months ago with FSH levels > 45 mU/mL and estradiol levels < 20 pg/mL
[41]	Premenopausal women (N = 243) followed over 16 years^1^ 35 to 47 years old at enrollment	52.7 % White 47.3 % Black/African American	ACE status and menopause stage	Incidence of MDD	Women reporting ≥ 2 ACEs were at significantly greater risk for lifetime and menopause MDD compared to those with 0 ACEs. Women reporting ≥ 2 ACEs during the postpubertal period, but not prepubertal, were 2.3x more likely to have menopause MDD.	Used STRAW+ 10 criteria to define menopause transition stages
[43]	Women Veterans (N = 70,864) Aged 55 + with ≥ 1 documented MST	63.5 % White 9.8 % Black/African American 0.4 % Hispanic 0.4 % Asian 25.8 % Other/Unknown	MST screening status	Menopause symptoms and odds of depression, anxiety, and PTSD	MST was associated with 7.25 times the odds of PTSD and over twofold odds of depression and suicidal ideation, as well as increased odds of anxiety, alcohol use disorder, substance use disorder, and opioid use disorder. A positive MST screen was associated with greater odds of obesity, chronic pain, back pain, insomnia, and sleep apnea.	Included women aged 55 and older, likely postmenopausal, but specific assessment not mentioned
[40]	Premenopausal, perimenopausal (early and late) and postmenopausal women (N = 142) followed over 16 years^1^ Mean baseline age of 45 years	53.9 % White 46.1 % Black/African American	ACE/stressful life event status and inflammatory marker levels	Depression symptom severity	Among those with high ACEs and a high number of current life stressors, higher levels of hsCRP were associated with higher odds of experiencing clinically significant depression symptoms.	Used STRAW+ 10 criteria to define menopause transition stages
[45]	Women (N = 6093) 18 to 65 years old < 40 years: premenopausal 40 – 55 years: perimenopausal > 55 years: postmenopausal	2.5 % White 93.8 % Black/African American 3.8 % Other (Hispanic/Latino, Asian, Multiracial)	Menopause stage (pre, peri, post)	PTSD and depression symptom severity	Perimenopausal women showed higher depression and PTSD symptom severity than premenopausal and postmenopausal women.	Used age cutoffs to categorize women as pre, peri, or postmenopausal
[39]	Women (N = 97) 40 to 65 years old	Not Available	CTQ status	Depression and menopause symptom severity	ACEs were highly prevalent among women seeking care for bothersome menopausal symptoms (66 %). Higher CTQ scores were significantly associated with higher depression scores, as well as with a greater burden of menopausal symptoms.	Included women attending a menopause clinic, but specific assessment not detailed
[44]	Women Veterans (N = 232) 45 to 64 years old	72.8% White 10.3 % Black/African American 3.9 % Asian 0.4 % American Indian/Alaskan Native 1.7 % Native Hawaiian/Pacific Islander 12.1 % Other	MST screening status	VMS, vaginal symptoms, sleep difficulty, depressive symptoms, anxiety symptoms, and PTSD symptoms	MST was associated with the presence of VMS, vaginal symptoms, insomnia, clinically significant depressive symptoms, anxiety, and PTSD.	Included women aged 45 –64, but specific menopause assessment not mentioned

1 Perimenopausal and postmenopausal status categorized using Stages of Reproductive Aging Workshop + 10 criteria (STRAW+10).

Lifetime trauma exposure was associated with greater instances of anxiety and depression in women of menopausal age. Perimenopausal women reported greater PTSD and depression symptoms. ACE: Adverse Childhood Experiences. CM: Childhood Maltreatment. CTQ: Childhood Trauma Questionnaire. FSH: Follicle-Stimulating Hormone. hsCRP: High-Sensitivity C-Reactive Protein. MD: Major Depression. MDD: Major Depressive Disorder. MDE: Major Depressive Episode. MST: Military Sexual Trauma. PTSD: Posttraumatic Stress Disorder. VMS: Vasomotor Symptoms.

**Table 2:** Summary of current literature analyzing the impact of trauma exposure on menopause onset and symptomatology.

Reference	Sample/Age	Racial-Ethnic Demographics	Trauma Type/Independent Variable	Outcome Measure	Results	Assessment of Menopause Stage

[55]	Perimenopausal and postmenopausal women^a^ (N = 295) 40 to 60 years old	73.22 % White 26.78 % Non-white	CTQ status	VMS during wake and sleep	Childhood sexual or physical abuse was associated with 1.5-two-fold the number of physiologically recorded VMS during sleep.	Used STRAW+ 10 criteria to define menopause transition stages
[42]	Women with one year of amenorrhea (N = 120) 40 to 65 years old	62.1 % White 37.1 % Black/African American 0.8 % Asian	Violence exposure status	Menopause symptom severity and rate of comorbid disorders such as osteoporosis, depression/psychological disorders, hypertension, rheumatic disease, allergies, fibromyalgia, varicose veins in the legs, labyrinthitis, diabetes, herniated disk, cancer	Women who experienced violence had a higher number of self-reported comorbidities during menopause (average of 4 –5) compared to 2.2 comorbidities in the control group. Total trauma exposure was associated with greater menopausal symptom severity. Sexual violence specifically was associated with poorer sexual health outcomes, but not with other menopausal symptoms.	Focused on postmenopausal women, defined as those who had their last menstrual period at least 12 months ago with FSH levels > 45 mU/mL and estradiol levels < 20 pg/mL
[51]	Women (N = 2016) 40 to 80 years old	39.4% White 21.3% Black/African American 20% Hispanic 19.2% Asian	Lifetime physical or emotional interpersonal violence, sexual assault, and current symptoms of PTSD	Menopause symptom severity	Symptoms of PTSD and emotional interpersonal violence were associated with difficulty sleeping, VMS, and vaginal symptoms. Physical interpersonal violence was associated with VMS and sexual assault was associated with vaginal symptoms.	Included women aged 40 –80, but specific menopause assessment not mentioned
[56]	Women Veterans (N = 232) 45 to 64 years old	74.1% White 10.3% Black/African American 3.9% Asian 1.7% Native Hawaiian 3.9% American Indian 3.4% Other	Lifetime IPV	Current clinical insomnia via the Insomnia Severity Index	Lifetime history of IPV was associated with twofold to fourfold odds of current clinical insomnia, including overall, physical, psychological, and sexual IPV.	Included midlife women veterans, but specific menopause assessment not mentioned
[52]	Women (N = 1670) 40 to 65 years old	93% White	ACE status	Menopause symptom severity	The authors observed a significant association between severe menopausal symptoms and higher childhood adversity (ACE score 1 –3 or ≥ 4 vs. ACE = 0). No association between ACEs and age of natural menopause observed.	Included midlife women, but specific menopause assessment not mentioned
[50]	Postmenopausal women (N = 350) Mean age of 59.2 years	92.9% White	ACE status	Timing of natural menopause		Menopause stage was determined by a medical provider using menstrual bleeding patterns
[48]	Postmenopausal women (N = 118) 40 to 68 years old	Not Reported	Lifetime incidence and type of VAW-economic, psychological, sexual, or physical violence	Timing of natural menopause and severity of menopause symptoms	Violence exposure was associated with increased menopausal symptoms and poorer quality of life. Violence-exposed women reached menopause approximately 20 months earlier and 20.7% of these women developed premature ovarian insufficiency.	Specific menopause assessment not clear from the available information
[57]	Premenopausal (early and late) and postmenopausal women (N = 167) followed over 14 years^1^ Mean baseline age of 44 years	51.5% White 48.5% Black/African American	ACE status and menopause stage	Inflammation (cytokines), verbal memory	Advancing menopause stage was associated with worse performance on immediate verbal recall and delayed verbal recall. During perimenopause, higher ACE exposure was associated with worse immediate verbal recall at higher levels of TNF-α.	Used STRAW+ 10 criteria to define menopause transition stages
[49]	Registered nurses (N = 46,639) Aged 25 to 42 at enrollment in 1989 Followed for up to 26 years	92.9% White 0.9% Black/African American 4.9% Other (Hispanic/Latino, Asian, Multiracial)	Trauma/PTSD Status	Timing of natural menopause or cessation of menses due to surgery and incidence of gynecologic surgeries	Women with trauma exposure, low, and high PTSD symptoms had earlier cessation of menses due to surgery but did not have earlier natural menopause than those without trauma exposure.	Used self-reported age at natural menopause
[53]	Women (N = 1572) 40 to 65 years old	96% White	ACE status	Sexual function	Women with a greater number of ACEs were more likely to be sexually inactive. Among sexually active women, the proportion of women with FSD increased sequentially as the number of ACEs increased.	Included midlife women, but specific menopause assessment not mentioned
[58]	Women (N = 40) 48 to 60 years old	75% White 17.5% Black/African American 5% Asian 2.5% Other	ACE status, TD	Functional network flexibility during a letter n- back working memory task	ACEs were associated with lower within-network connectivity while TD had no effect on connectivity in the low ACE group. TD increased connectivity in the high ACE group. Lower within network connectivity was associated with poor performance on the CPT attention task.	Included hypogonadal women based on menstrual cycle changes with FSH levels > 30 IU/mL
[59]	Women (N = 33) 48 to 60 years old	75.8% White 18.2% Black/African American 6% Asian	ACE status, tryptophan depletion and, estradiol treatment	Brain activation during working memory task in menopausal women	In the absence of exogenous estradiol, TD increased right DLPFC activation in high ACE subjects but decreased activation in low ACE subjects. Treatment with estradiol attenuated the effects of ACE and TD.	Included early hypogonadal women based on menstrual cycle changes with FSH levels > 30 IU/mL
[60]	Healthy hypogonadal women (N = 40) 48 to 60 years old	Not Reported	ACE status, TD	Functional network flexibility during a letter n-back working memory task	ACEs were associated with higher flexibility across networks. There was no interaction between ACE and TD, however TD increased network flexibility in both ACE groups in comparison to sham. depletion. TD was associated with worse CET performance, however there was no significant association of ACE status on CET performance.	Specific menopause assessment not clear from the available information
[39]	Women (N = 97) 40 to 65 years old	Not Available	CTQ status	Depression and menopause symptom severity	ACEs were highly prevalent among women seeking care for bothersome menopausal symptoms (66%). Higher CTQ scores were significantly associated with higher depression scores, as well as with a greater burden of menopausal symptoms.	Included women attending a menopause clinic, but specific assessment not detailed
[54]	Women (N = 332) interviewed annually over eight years 42 to 52 years old	67% White 33% Black/African American	CTQ status	VMS	Childhood abuse or neglect was associated with increased reporting of hot flashes and night sweats.	Used data from the Study of Women’s Health Across the Nation (SWAN), which defined menopausal transition stages based on menstrual bleeding patterns

a Perimenopausal and postmenopausal status categorized using Stages of Reproductive Aging Workshop + 10 criteria (STRAW+10).

Greater trauma history was associated with greater menopausal symptom burden but mixed effects on menopause onset. Childhood trauma was associated with poor cognitive outcomes during menopause. ACE: Adverse Childhood Experiences. CET: Conditional Exclusion Test. CPT: Penn Continuous Performance Task. CTQ: Childhood Trauma Questionnaire. DLPFC: Dorsolateral Prefrontal Cortex. FSD: Female Sexual Dysfunction. FSH: Follicle-Stimulating Hormone. IPV: Intimate Partner Violence. PTSD: Posttraumatic Stress Disorder. TD: Tryptophan Deletion. VAW: Violence Against Women. VMS: Vasomotor Symptoms.
